# Identification of an early assembly factor for photosystem II biogenesis

**DOI:** 10.1093/plcell/koae220

**Published:** 2024-07-24

**Authors:** Renuka Kolli

**Affiliations:** Assistant Features Editor, The Plant Cell, American Society of Plant Biologists; Sainsbury Laboratory, University of Cambridge, Cambridge, UK

**Figure 1.**
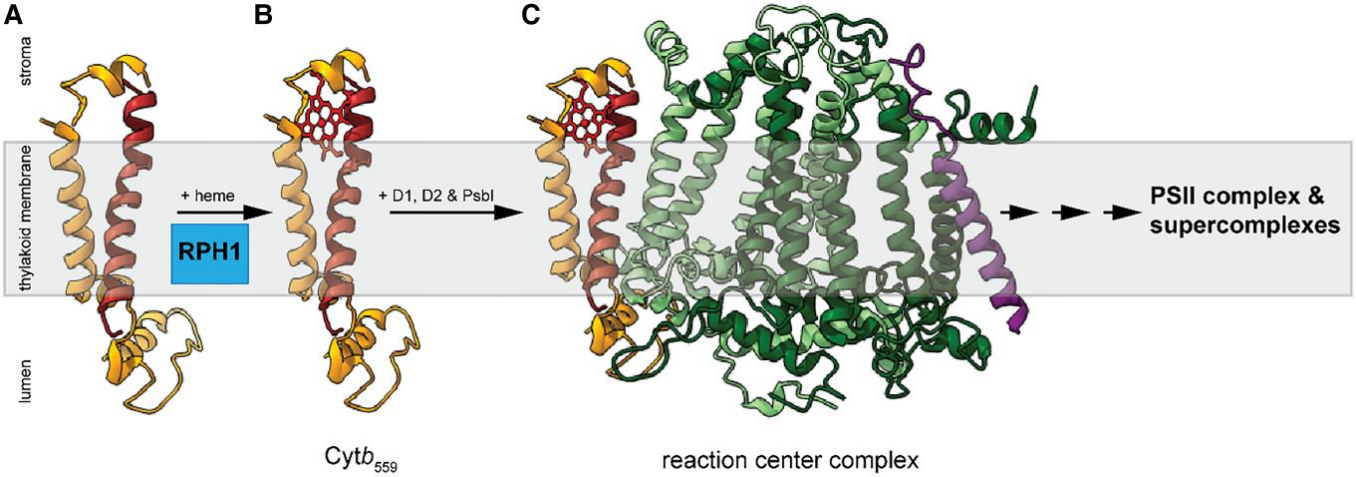
Early PSII assembly pathway. **A)** Apo-Cyt*b*_559_. **B)** Cyt*b*_559_. **C)** The reaction center complex goes through multiple assembly steps with the addition of other protein subunits and cofactors to form the PSII complex and then the supercomplexes. The heme is shown in red, and the protein subunits are colored as follows: α subunit, orange; β subunit, brown; D1, dark green; D2, light green; PsbI, purple. The cofactors bound by D1, D2, and PsbI are not shown for simplicity's sake. Figure credit: R. Kolli. The Arabidopsis PSII supercomplex structure obtained from the PDB database (https://www.rcsb.org/structure/5MDX) was adapted using ChimeraX (Meng et al. 2023).

Photosystem II (PSII) initiates photosynthesis in oxygen-evolving photosynthetic organisms, including cyanobacteria, algae, and plants, by catalyzing light-driven electron transfer from water to plastoquinone. PSII is a large thylakoid membrane–localized complex composed of at least 20 protein subunits and many cofactors, including chlorophylls, hemes, quinones, and the unique Mn_4_CaO_5_ cluster. Its homodimer forms supercomplexes with a variable number of light-harvesting complexes II depending on the light intensity. Cytochrome *b*_559_ (Cyt*b*_559_) is an essential component of PSII required for PSII assembly and function, likely by participating in secondary electron transfer pathways and preventing photoinhibition (Chiu and Chu 2022). The reaction center complex, consisting of D1, D2, Cyt*b*_559_, and PsbI, is the first transiently accumulating complex during the early phase of PSII biogenesis (Nickelsen and Rengstl 2013; Fig. 1C). Cyt*b*_559_ is a heme-bridged heterodimer of the α and β subunits, encoded by the chloroplast genes, *psbE* and *psbF*, respectively (Fig. 1B). The heme is noncovalently bound by a histidine residue from each of the subunits. Although numerous studies have been conducted on the structure and function of Cyt*b*_559_, its assembly pathway and the exact function in oxygenic photosynthesis remain enigmatic.

In this issue, **Li-Ping Che and coauthors** (Che et al. 2024) show that *Arabidopsis thaliana* RPH1 is an assembly factor of Cyt*b*_559_ and is thereby crucial for PSII assembly. RPH1 was originally identified as a conserved chloroplast protein required for plant disease resistance against oomycete pathogens, such as *Phytophthora brassicae* and the late blight-causing *Phytophthora infestans* (Belhaj et al. 2009). Che et al. found that PSII abundance is severely reduced in *rph1* mutants compared with the wild type, likely a causal factor in their lower photosynthetic activity and drastic retardation in plant growth and development. Based on immunoblot analyses and pulse-chase labeling, the authors attribute the reduction in PSII abundance in the *rph1* mutants to a defect in Cyt*b*_559_ formation. The authors ruled out alterations in *psbE* and *psbF* gene expression and protein synthesis in *rph1* mutants as the transcript levels and ribosome occupancies were similar to the corresponding wild-type levels.

A mutant of the α subunit in the green alga *Chlamydomonas reinhardtii* can no longer bind heme to form Cyt*b*_559_ but can still assemble PSII supercomplexes to 15% to 20% of the wild-type levels (Hamilton et al. 2014). As the PSII supercomplexes in *rph1* are similarly reduced, Che et al. measured the heme level from the dithionite-reduced minus ferricyanide-oxidized optical difference spectra. The heme level of *rph1* PSII supercomplexes was severely reduced compared with that of wild-type PSII supercomplexes. Hence, RPH1 appears to facilitate heme insertion during Cyt*b*_559_ formation. Accordingly, localization studies indicated RPH1 to be an intrinsic thylakoid membrane protein with 4 predicted transmembrane helices, and the N-terminus was found to be exposed to the stroma based on protease digestion of the wild-type thylakoids followed by immunoblotting. Moreover, RPH1 directly interacts with both the Cyt*b*_559_ subunits, as evident from split-ubiquitin 2-hybrid assays and bimolecular fluorescence complementation. Interestingly, apo-Cyt*b*_559_ was specifically detected in the absence of RPH1 (Fig. 1A). Therefore, the authors propose that RPH1 is a dedicated factor for facilitating non-covalent heme incorporation during Cyt*b*_559_ formation in etioplasts and chloroplasts. Furthermore, they emphasize the central physiological role of Cyt*b*_559_ in plant photoprotection by showing that under photoinhibitory conditions, the *rph1* mutant has higher photosensitivity and elevated levels of reactive oxygen species in comparison with the wild-type and other PSII mutants investigated.

In summary, RPH1, initially identified to be required for pathogen resistance, has now been found to be a putative molecular chaperone for heme insertion into apo-Cyt*b*_559_ to form the functional holo-Cyt*b*_559_ during PSII assembly in Arabidopsis chloroplasts (Belhaj et al. 2009; Che et al. 2024; Fig. 1). This key finding paves the way for uncovering the mechanism of heme insertion during Cyt*b*_559_ biogenesis and further exploration of its essential role in PSII photoprotection and its relationship to plant immunity.
